# Topics of Public Interest

**Published:** 1908-01

**Authors:** 


### TOPICS OF PUBLIC INTEREST.

### No Disease On Money.

**Doctor Doty's Opinion — Foolish to Consider it Medium of Transmission, He Says**

DR. A. H. DOTY, Health Officer of this port, who for years has made a study of infectious diseases, and especially the medium of their transmission, does not agree with the theorists who contend that money is a transmitter of disease.

Dr. Doty said recently that, while bacteriological examinations were presented to show that different forms of bacteria were found on money, practical and careful observation had proved that infection was caused and epidemics were spread in nearly all instances by personal contact with infected persons, and more especially with ambulant cases, rather than through the handling of money and clothing. He says, moreover, that too much needless and harmful agitation is being devoted to the theory that money is an agent of disease. He contends that there are many other considerations in the protection of public health that constitute a real menace that should take up the consideration of the public rather than conditions which are based on theory. Concerning the current agitation on money as an agent of disease, Dr. Doty said:

The theory that money acts as a medium of infection is a plausible one, particularly as it deals with an agent which is being constantly transmitted from one person to another and among all classes of people. Clothing, rags, merchandise, cargoes of vessels, etc., are also regarded as means by which disease is commonly transmitted.

### WHERE BACTERIA ARE FOUND.

This belief is popular because it offers an explanation for outbreaks of infectious disease the origin of which is unknown. Modern sanitation, however, does not regard as valuable theories which are unsupported by fact or practical experience. The theory that money acts as a medium of infection carries with it no satisfactory or even reasonable proof. It is true that from time to time the results of bacteriological examinations are presented to show that different forms of bacteria are found on money. No one who is familiar with the subject doubts this, but the same organisms may at almost any time be found on our hands, on stair railings and all exposed places. These bacteria are, as a rule, harmless, and some of them are a benefit to mankind. Even from a bacteriological point of view there are reasons why money would not be likely to transmit disease. However, this question must be decided principally by reliable statistics and the results of practical experience.

### HANDLING MONEY IN BULK.

Whoever may be inclined to investigate this subject in a reasonable way and will visit the Treasury Department at Washington, where an enormous amount of old and filthy paper money is being constantly handled and rehandled, or will seek information from bank officials, will find that those who are connected with this work do not contract infectious disease any oftener than any one else.

There is no reason why persons thus employed may not contract infectious disease, because they are subjected to the same outside exposure that others are, but this furnishes no proof that money is the medium of transmission. It is rather to be regarded as a coincidence. There is probably no doubt that in rare instances money, like other things, may act as a means of transmitting disease, but it is so uncommon that we must not give it undue consideration, for there are so many other considerations with which we must deal in protecting the public health that constitute a real menace that we should rather devote our energies to these than to conditions which are based on theory. The fear that money transmits disease is, I am quite sure, largely due to the fact that it is frequently old and filthy. While this is unpleasant in many ways, it does not indicate the presence of pathogenic organisms—that is, the germs which transmit infectious disease.

### WORKERS AMONG RAGS.

Both domestic and foreign rags are also regarded as a medium of infection, and commerce has frequently been crippled by the detention and disinfection of vessels arriving from foreign ports having rags as a part of their cargoes. Still, we have no reasonable evidence that this material transmits disease. My own observation in regard to this part of the subject includes not only the collection of reliable statistics from paper manufacturers in this country, but also a personal investigation of the rag depots at Alexandria, Egypt. Here rags, consisting principally of worn-out clothing or gowns, are brought from all parts of lower Egypt in rope crates, and are sorted and resorted by women and children, but at the time of my visit no evidence had ever been presented to show that these ragpickers were more subject to infectious disease than those not connected with the work.

These statistics were carefully compiled by British sanitary officers who are charged with the protection of the public health in the country just referred to. The same results were obtained in the investigation of this subject in connection with paper manufacturers in the United States, where the bulk of foreign and domestic rags are used.

Careful investigation has, during the last few years, thrown much light on the subject of the transmission of disease and as a consequence many popular theories have been shattered. It was not very long ago that clothing was regarded as one of the common means by which yellow fever was transmitted. We know now that this disease is transmitted only by the mosquito, and that clothing has nothing whatever to do with it.

### MALARIA FROM MOSQUITO.

Until recently it was believed that malaria was caused by bad air, emanations from swamps, etc. Conclusive evidence, however, has been presented to show that this theory is incorrect, and that malaria is also transmitted by the mosquito.

Those who actually deal with infectious disease and carefully study the means by which it is transmitted learn that clothing, money, cargoes of vessels, etc., act as a medium of infection only in rare instances, and what we have most to fear in our efforts to prevent the appearance or extension of infectious disease is the presence of mild and unrecognised cases which often occur and which constitute one of the most dangerous factors with which public health officials are obliged to deal, and it is this which is commonly the cause of outbreaks, and which contributes support to the theory that money, etc., is a medium of infection. For, as these cases are not recognised, some theory must be advanced for the outbreak of infectious disease, the origin of which is not found. Those in charge of the public health are beginning to realise this fully and to know that one of the most important means of controlling an out-break of infectious disease is to cause a thorough and exhaustive inspection to be made for the purpose of detecting mild or ambulant cases.

### CAN'T DISINFECT MONEY.

It is fortunate that money constitutes such an unimportant factor in the transmission of disease, as nothing could be more farcical from a sanitary point of view than an attempt to disinfect it, although this has been seriously proposed.

It is important that the public should know the advances which from time to time are made in sanitary science, that they may intelligently coöperate with health officials, particularly in emergencies, for there is nothing which contributes more to a successful result in dealing with outbreaks of infectious disease than the aid which the public may extend at these times.

---

THE ACTION OF THE RADIANT LIGHT BATH IN NERVOUS DISEASES.—T. D. Crothers, of Hartford, gives the results of ten years' use of the radiant light bath in the treatment of nervous diseases of the sclerotic type, fibrosis, local irritations, and inflammatory states of subacute nature. The radiant light bath is a powerful sudorific, acting much more rapidly than hot air. It also has some specific effect on the cells and tissues, is an eliminant, influences metabolism and nutrition, and its physical effects on the nervous system affect the mind and emotions. Sweating begins in a few minutes. Insomania and exhilaration are followed by a deep and refreshing sleep. It overcomes nervousness and irritation in drug-takers. It diminishes the desire to take spirits and drugs. Appetite is stimulated and digestion improved. Thirst, relaxation of the bowels, and renal activity are results that may occur. The action of bromides is increased by the bath. Mental relief and buoyancy of mind are remarkable. Despondency passes away and restfulness follows. Arterial tension is diminished.—*Medical Record*, November 23, 1907.

